# *Weissella koreensis* regulates mitochondrial protective genes and innate immune genes to enhance anti-bacterial immunity

**DOI:** 10.3389/fimmu.2026.1998836

**Published:** 2026-10-21

**Authors:** Zeyu Hou, Wenting Fu, Rui-Qiu Yang, Fang Liu, Yi Xiao

**Affiliations:** 1 Department of General Surgery, Affiliated Hospital of Zunyi Medical University, Zunyi, Guizhou, China; 2 Department of Thyroid and Breast Surgery, Affiliated Hospital of Zunyi Medical University, Zunyi, Guizhou, China; 3 College of Basic Medicine, Zunyi Medical University, Zunyi, Guizhou, China; 4 Institute of life Sciences, Zunyi Medical University, Zunyi, Guizhou, China; 5 The Key Lab of Guizhou Provincial Department of Education for Medical Prevention and Treatment of Tumor, Zunyi Medical University, Zunyi, Guizhou, China; 6 School of Basic Medicine, Kunming Medical University, Kunming Yunnan, China

**Keywords:** antimicrobial peptide, *Caenorhabditis elegans*, innate immunity, mice, mitochondrial unfolded protein response

## Abstract

Probiotics are widely recognized for their ability to promote gut health, modulate immunity, and reduce oxidative stress. As an emerging probiotic within the genus *Weissella*, *Weissella koreensis* (WK) exhibits promising immunomodulatory potential. Recent evidence highlights its potential as a functional probiotic in combating obesity. However, its specific role in host immune regulation and the underlying molecular mechanisms remain largely undefined. In this study, we demonstrate that, compared to the OP50-fed controls, WK significantly increases resistance to not only the Gram-negative pathogens *Pseudomonas aeruginosa* and *Salmonella enterica* but also the Gram-positive pathogens *Staphylococcus aureus* and *Enterococcus faecalis* by promoting intestinal bacterial clearance. Through genetic analysis in *Caenorhabditis elegans*, we find that WK promotes innate immunity via the transcription factor ATFS-1. We further show that WK activates the mitochondrial unfolded protein response (UPR^mt^) and downstream innate immune effector genes through ATFS-1. In addition, WK decreases reactive oxygen species (ROS) levels and increases adenosine triphosphate (ATP) levels in *C. elegans*. Consistent with a potentially conserved host response, WK treatment in mice improved survival, reduced pulmonary bacterial burden, and increased the transcription of *Atf5* and associated mitochondrial-protective genes. These findings advance our understanding of the immunomodulatory functions of *Weissella koreensis* and highlight its efficacy as a novel therapeutic strategy for immune intervention.

## Introduction

Innate immunity is the frontline defense against microbial infections and is evolutionarily conserved across species, spanning from nematodes to mammals (1). During infection, it rapidly initiates immune responses to eliminate invading pathogens while preserving organismal homeostasis (1, 2). *Caenorhabditis elegans* (*C. elegans*) has been developed as an ideal genetic model for studying immunity and its resistance to *P. aeruginosa* PA14 directly reflects the efficacy of immune function (3). Several signaling pathways that play important roles in innate immunity have been identified, including the DAF-2/DAF-16 signaling pathway (4, 5), p38 mitogen-activated protein kinase (MAPK)/PMK-1 pathway (6, 7), extracellular signal-regulated kinase (ERK) MAPK/MPK-1 (8), endoplasmic reticulum unfolded protein response (UPR^ER^)/XBP-1 (9, 10) and mitochondrial unfolded protein response (UPR^mt^) mediated by ATFS-1 (11, 12).

In recent years, probiotics have been defined as live microorganisms that confer health benefits to the host when administered in adequate amounts (13). They are generally categorized into four major groups, namely *Lactobacillus*, *Bifidobacterium*, *Streptococcus* and *Enterococcus*. Among beneficial microbes, the genus *Weissella*, an important group of lactic acid bacteria capable of colonizing the gastrointestinal tract (14), has garnered increasing attention. Due to striking physiological similarities, the *Weissella* genus was originally classified within the family *Leuconostocaceae (*15). This lineage was recognized as a distinct and independent genus, named in honor of the German microbiologist Norbert Weiss (16). The *Weissella* genus possesses many health benefits and functions, including anti-tumor effects (17), anti-obesity properties (18, 19), lifespan-extending capabilities (20) and immune-regulating effects (21). *Weissella koreensis* (WK), a species within the genus *Weissella*, is predominantly isolated from traditional fermented foods and has been investigated primarily for its fermentation characteristics (22, 23). Moreover, there is evidence that it has potential as a functional probiotic in immune regulation (24). Previous studies have shown that it played an important role in combating obesity (18, 19) and could significantly extend the basal lifespan of *C. elegans (*20). Despite these known benefits in promoting general healthspan, it remains poorly understood whether WK plays a specific role in the host’s immune mechanism, nor is the underlying molecular mechanism fully defined. Clarifying the specific role and targets of WK in innate immunity will expand current knowledge of its biological functions, providing a theoretical foundation for developing WK as a novel immunomodulatory probiotic.

Mitochondria play essential roles in antibacterial innate immunity including UPR^mt^ activation, reactive oxygen species (ROS) production, and mitophagy (11, 25, 26). When mitochondrial stressors are present, UPR^mt^ enhances mitochondrial protein homeostasis through transcriptional control of nuclear-encoded chaperones and proteases, a process orchestrated by the ATFS-1 transcription factor in *C. elegans (*27, 28). In mammals, early research established that activating transcription factor 5 (ATF5) serves a similar role as ATFS-1 in *C. elegans*, both of which are evolutionarily conserved in activating the UPR^mt29^. Previous studies have shown this conserved pathway and its key regulator ATFS-1 upregulate not only mitochondrial protective genes but also immune effector genes, including lysozymes and antimicrobial peptides (12, 27).

In this study, we investigated the role of *Weissella koreensis* in the host defenses of *C. elegans* and mouse models. We demonstrated that WK significantly increased the resistance of both nematodes and mice to *P. aeruginosa* PA14 infection. In addition, WK promoted innate immunity through the activation of UPR^mt^ and immune-related genes in an ATFS-1-dependent manner in *C. elegans*, while inducing the transcriptional upregulation of the homologous ATF5-associated UPR^mt^ program in mice. These findings bridge a gap in understanding the immunomodulatory functions of *Weissella koreensis*, elevating its application from a traditional food fermentation agent to a highly promising functional probiotic for therapeutic immune interventions.

## Results

### *Weissella koreensis* colonizes the *C. elegans* intestine and enhances host resistance to bacterial infections

To test whether *Weissella koreensis* impacts innate immunity, worms were challenged with the human opportunistic pathogen *Pseudomonas aeruginosa* (PA14). We found that WK treatment significantly prolonged the survival of wild-type *C. elegans* following *P. aeruginosa* PA14 exposure compared with the OP50-fed group. The results showed that WK-treated worms exhibited increased resistance to PA14 (**Figure 1A**), indicating that WK can enhance innate immunity in *C. elegans*. After 3 days of feeding, we observed that green fluorescent protein (GFP) -labeled WK was readily detected in the intestinal tract of *C. elegans* (**Figure 1B**), showing WK is able to colonize the host intestine. To determine whether live WK bacteria can achieve true colonization in the nematode intestine, after feeding the worms with WK, we tested the accumulation of WK in the worms’ intestines at 3, 6, 9 days. We observed the enrichment of WK in the intestinal tract of the nematode (**Figure 1C**). Since reducing intestinal bacterial load plays an important role in the host defense strategy against pathogen infection (29), we tested whether WK influenced the bacterial accumulation in the nematode intestine. We discovered WK could reduce the intestinal bacterial burden in PA14-infected wild-type (N2) worms for 48 hours compared with OP50-fed group (**Figure 1D**). We found that WK did not influence the pumping rate of *C. elegans* (**Figure 1E**). To assess whether intestinal colonization by WK really enhances the host defense against invading pathogens, we exposed worms to *Salmonella enterica*, *Staphylococcus aureus*, and *Enterococcus faecalis*. In each case, administration of WK markedly prolonged the survival of *C. elegans* compared with the OP50-fed controls (**Figures 1F–H**). Our results demonstrate that WK reduces the intestinal pathogen burden, thereby enhancing host resistance to bacterial infection in *C. elegans*.

**Figure 1:**
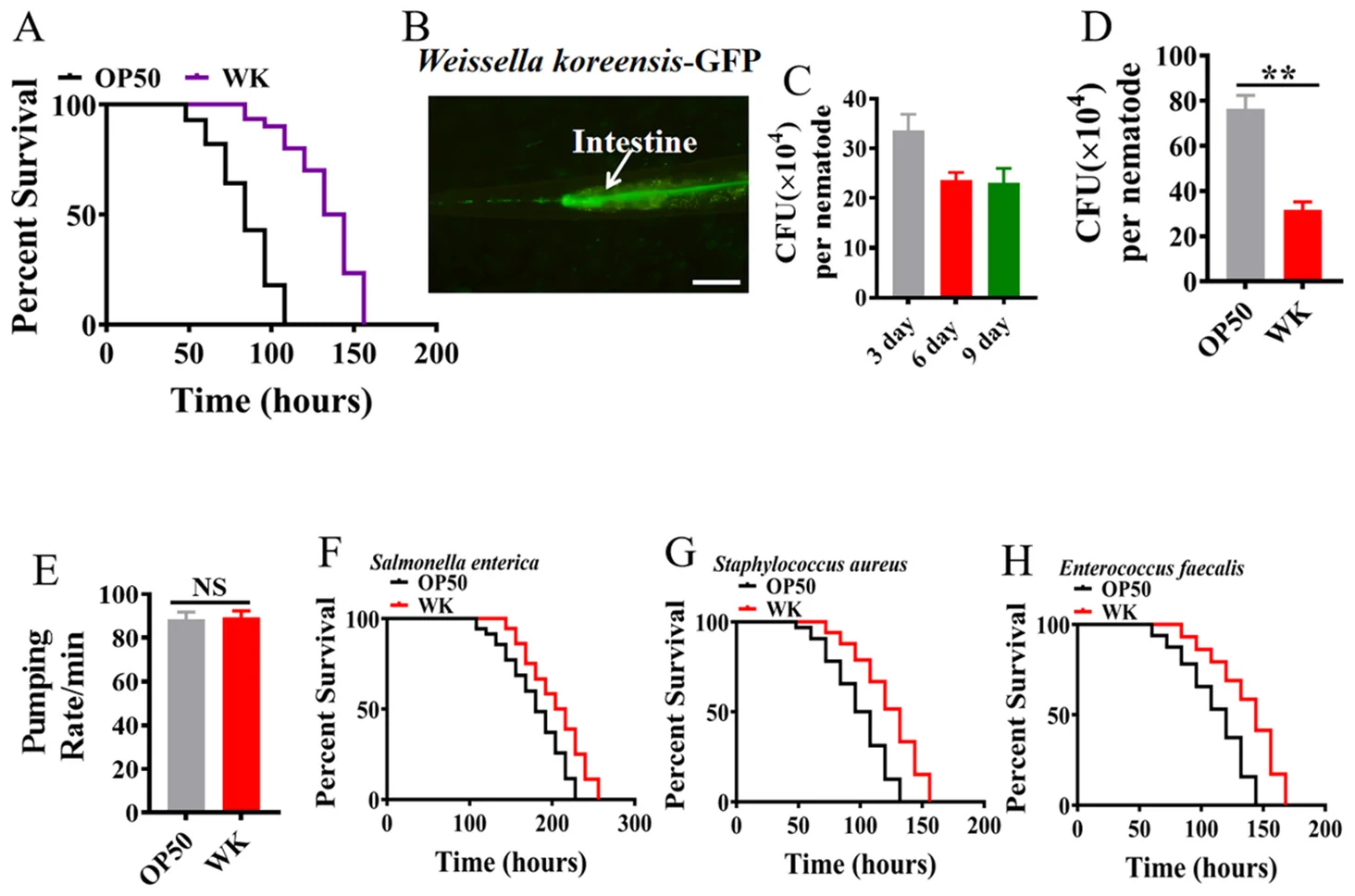
*Weissella koreensis* colonizes the *C. elegans* intestine and enhances host resistance to bacterial infections. **(A)** Survival curves of wild-type *C. elegans* exposed to *P. aeruginosa* PA14 following pretreatment with OP50 or *Weissella koreensis* (WK). (*P < 0.05; log-rank test). (n ≥ 40). **(B)** Representative fluorescence image showing intestinal colonization of GFP-labeled WK in *C. elegans* after 3 days of feeding. Scale bar: 50 μm. **(C)** Intestinal bacterial burden of WK in wild-type worms at 3, 6, 9 days. **(D)** Intestinal bacterial burden of *P. aeruginosa* PA14 in wild-type worms at 48 h after infection. (**P < 0.01, unpaired t-test) (n ≥ 10). **(E)** Pumping rate of wild-type *C. elegans* exposed to *P. aeruginosa* PA14 following pretreatment with OP50 or *Weissella koreensis* (WK). Data are presented as mean ± SEM from three independent biological replicates. NS (no significance). **(F–H)** Survival curves of *C. elegans* infected with **(F)** *Salmonella enterica*, **(G)** *Staphylococcus aureus*, **(H)** *Enterococcus faecalis* (*P < 0.05; log-rank test) (n ≥ 40).

### *Weissella koreensis* enhances innate immunity through ATFS-1

To clarify the molecular mechanism underlying *Weissella koreensis*-mediated protection against bacterial infection, we screened several classical signaling pathways involved in the innate immune response of *C. elegans*, including the DAF-2/DAF-16 signaling pathway (4, 5), p38 MAPK/PMK-1 pathway (6, 30), ERK MAPK/MPK-1 (8), UPR^ER^/XBP-1 (9, 31) and mitochondrial unfolded protein response (UPR^mt^) mediated by ATFS-1 (11, 12). We then evaluated whether WK retained its protective effect in mutants defective in each of these pathways following *P. aeruginosa* PA14 infection. Interestingly, we found WK still prolonged the survival of wild-type (N2), *daf-2(e1370)*, *pmk-1(km25)*, *mpk-1(n2521)*, and *xbp-1(zc12)* mutants compared with the respective OP50-fed controls (**Figures 2A–E**). However, we observed WK failed to prolong the survival of the *atfs-1(gk3094)* mutant compared with OP50-fed group (**Figure 2F**). Overall, these results indicate that the ATFS-1-mediated UPR^mt^ pathway is required for WK-induced resistance to bacterial infection.

**Figure 2:**
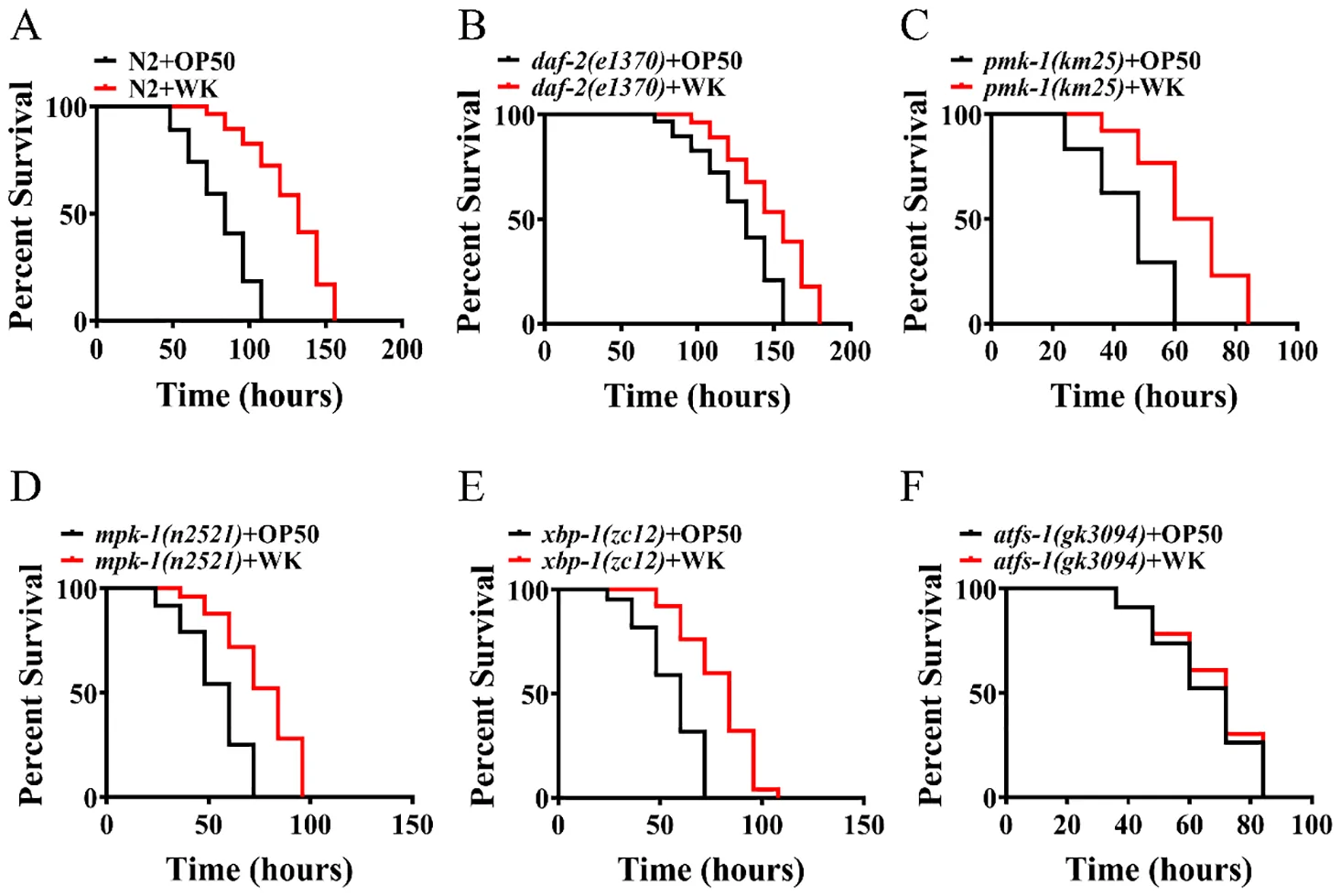
*Weissella koreensis* improves innate immunity through ATFS-1. Survival curve of WT (N2) **(A)**, *daf-2(e1370)* **(B)**, *pmk-1(km25)* **(C)**, *mpk-1(n2521)* **(D)**, *xbp-1(zc12)* **(E)**, and *atfs-1(gk3094)* **(F)** mowms exposed to *P. aeruginosa* PA14 following feeding with OP50 or WK. (*P < 0.05; log-rank test) (n ≥ 40).

### *Weissella koreensis* activates ATFS-1-dependent UPR^mt^ to preserve mitochondrial homeostasis and promote bacterial clearance

We hypothesized that WK maintains mitochondrial homeostasis by activating the UPR^mt^. To link this ATFS-1-mediated response directly to bacterial clearance, we monitored intestinal bacterial load in *atfs-1(gk3094)* mutants. Notably, compared to the OP50-fed controls, WK significantly cleared the intestinal bacterial burden in wild-type worms by 48 hours post-PA14 infection, but lacked this clearance capacity in *atfs-1(gk3094)* mutant worms (**Figure 3A**). Meanwhile, we found that WK did not influence the pumping rate in WT and *atfs-1(gk3094)* mutant worms (**Supplementary Figure S1**). Whether WK activated the transcription factor ATFS-1, we tested the cellular translocation of ATFS-1 using transgenic worms that expressed a functional ATFS-1::GFP fusion protein. We found that WK significantly induces ATFS-1 nuclear localization in the intestine (**Figure 3B**). Quantitative real-time PCR (qRT-PCR) also confirmed a robust upregulation of *atfs-1* mRNA expression in WK-treated animals versus the controls (**Figure 3C**). Functionally, UPR^mt^ activation was accompanied by decreased ROS levels and increased the adenosine triphosphate (ATP) levels, indicating improved mitochondrial function. Subsequent quantification revealed that WK treatment markedly suppressed ROS accumulation while elevating ATP production compared to the control groups. In contrast, WK failed to change the levels of ROS and ATP in *atfs-1(gk3094)* mutant worms (**Figures 3D, E**). Moreover, qRT-PCR analysis showed that WK upregulated the transcription of classic ATFS-1-targeted mitochondrial protective genes *hsp-6* and *hsp-60* compared with the control. In contrast, WK failed to increase the expression of these genes in *atfs-1(gk3094)* mutant worms (**Figure 3F**). To further verify the response, we monitored the *hsp-60::GFP* reporter and found that WK induced its expression in an ATFS-1-dependent manner (**Figures 3G, H**). Furthermore, we found that heat-killed WK did not increase the expression level of *hsp-60::GFP* (**Supplementary Figure S2**). These findings suggest that *Weissella koreensis* preserves mitochondrial homeostasis and promotes bacterial clearance through ATFS-1-dependent UPR^mt^ activation.

**Figure 3:**
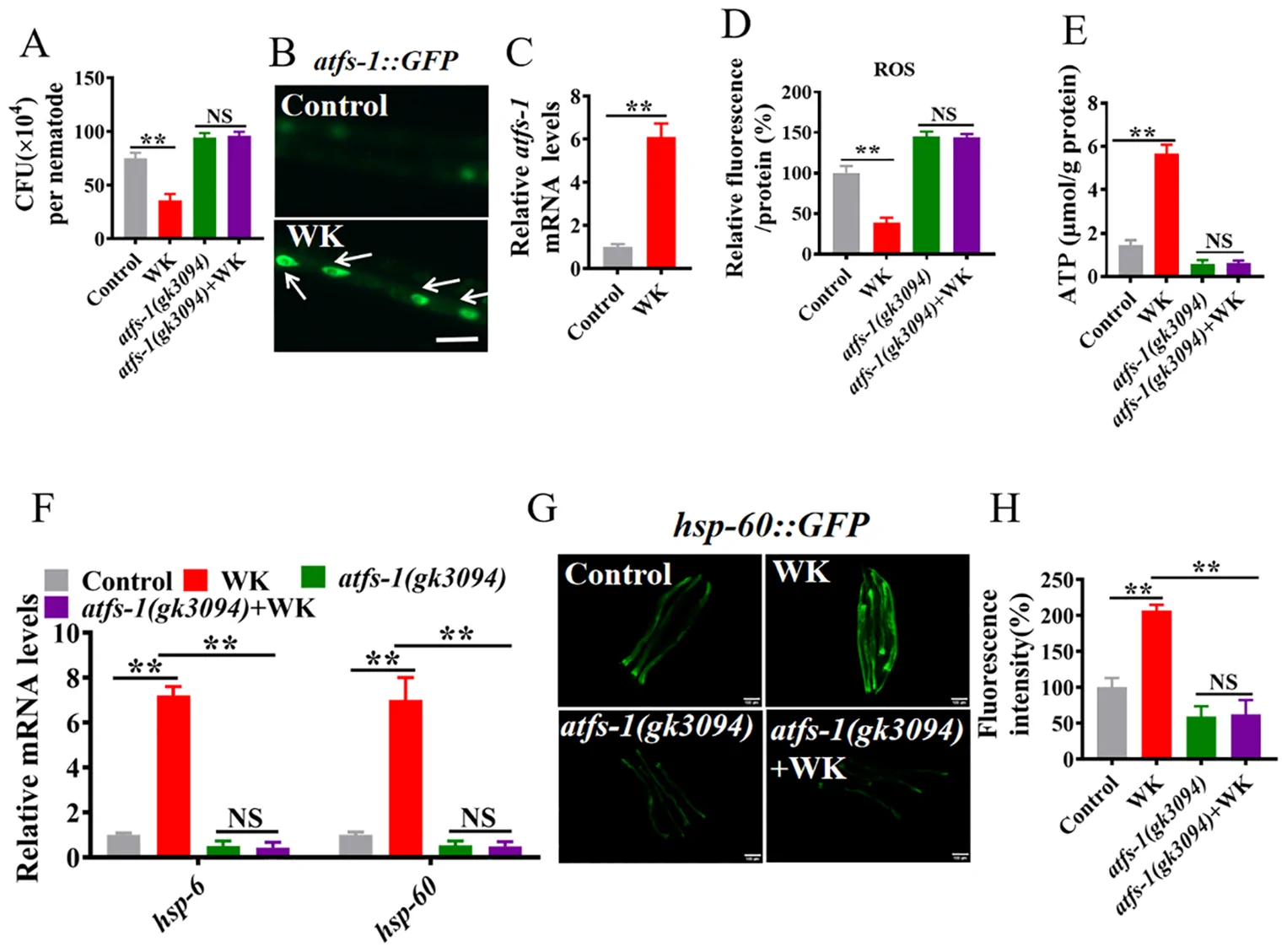
*Weissella koreensis* activates ATFS-1-dependent UPR^mt^ to preserve mitochondrial homeostasis and promote bacterial clearance. **(A)** Quantification of intestinal *P. aeruginosa* PA14 CFUs in wild-type and *atfs-1(gk3094)* worms at 48 h after infection. Mean ± SEM of three independent experiments. (**P < 0.01, one-way ANOVA). (n ≥ 10). **(B)** The cellular translocation of *atfs-1::GFP* following WK treatment. Scale bars: 50 μm. **(C)** Relative *atfs-1* mRNA expression following WK treatment. (**P < 0.01, unpaired t-test). **(D, E)** Intracellular ROS levels **(D)**, and ATP production **(E)** in wild-type and *atfs-1(gk3094)* mutant worms following WK treatment. (**P < 0.01, one-way ANOVA). **(F)** Relative mRNA levels of mitochondrial chaperones genes *hsp-6* and *hsp-60* in wild-type and *atfs-1(gk3094)* mutant worms. (**P < 0.01, one-way ANOVA). **(G)** Representative fluorescence images showing *hsp-60::GFP* reporter expression in wild-type and *atfs-1(gk3094)* mutant worms fed OP50 or WK. Scale bars: 100 μm. **(H)** Quantification of *hsp-60::GFP* fluorescence intensity shown in panel **(G)** (n ≥ 10). These results are mean ± SEM of three independent experiments performed in triplicate (**P < 0.01, one-way ANOVA).

### *Weissella koreensis* promotes antimicrobial peptide (AMP) expression through ATFS-1-dependent regulation of innate immune effector genes

Previous studies have shown that the host defense of *C. elegans* relies heavily on the expression of the innate immune effector genes, such as antimicrobial peptides (AMPs), which directly eliminate invading pathogens. Compared to the control, qRT-PCR analysis showed WK elevated the mRNA levels of ATFS-1-dependent immune response genes (*clec-65*, *clec-4*, *irg-1*, *ilys-2* and *abf-2*). But WK was not able to upregulate these genes in *atfs-1(gk3094)* mutant worms (**Figure 4A**). To further investigate the expression of these innate immune effector genes, we monitored the transcriptional activity of the *irg-1::GFP* and *ilys-2::GFP* reporters in *C. elegans (*30). WK induced the expression of both reporters. However, this effect was eliminated in *atfs-1(gk3094)* mutant worms (**Figures 4B, E**). These data indicate that WK enhances the expression of AMP genes to strengthen innate immunity.

**Figure 4:**
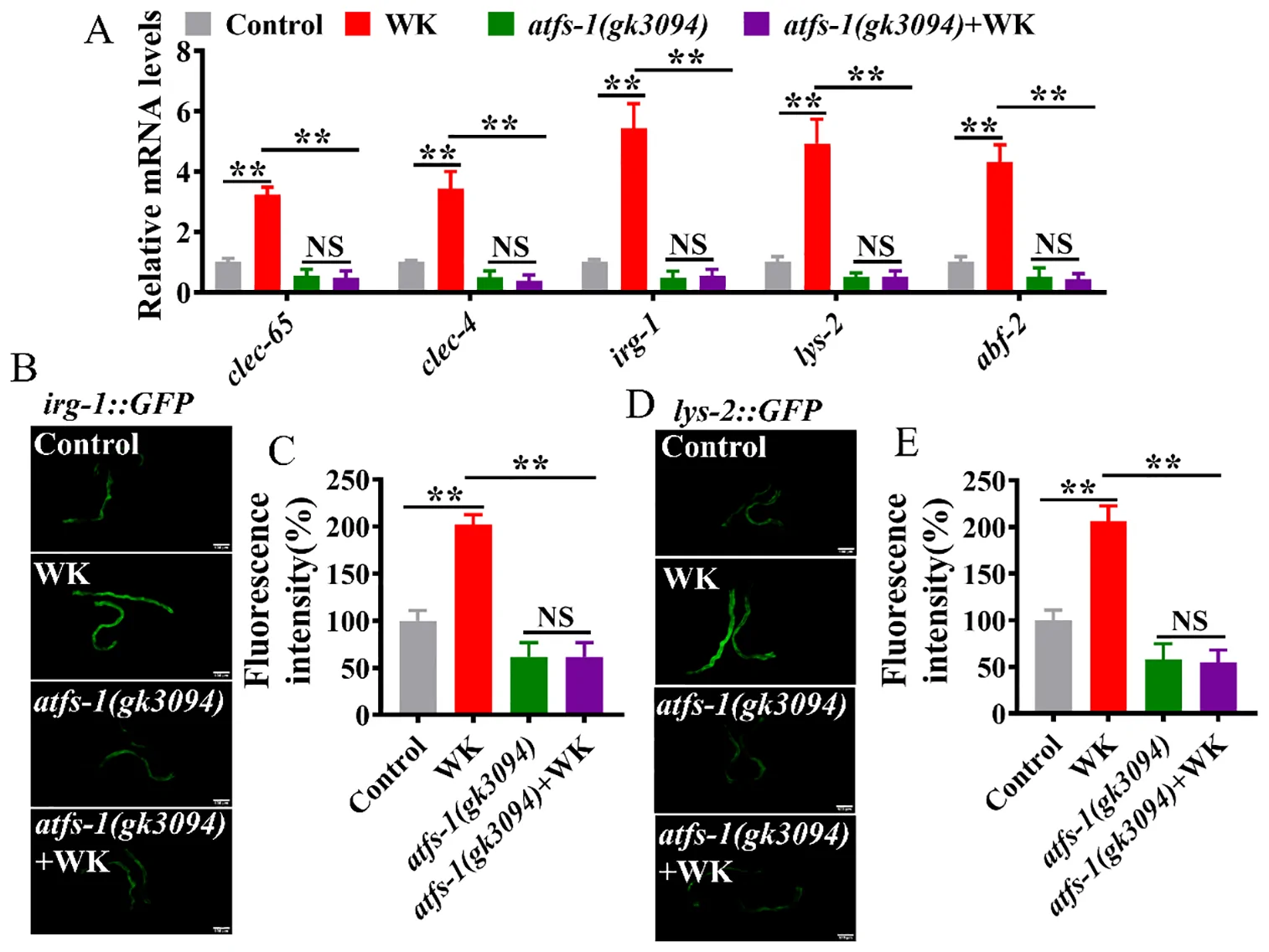
*Weissella koreensis* promotes antimicrobial peptide (AMP) expression through ATFS-1-dependent regulation of innate immune genes. **(A)** Relative mRNA expression of ATFS-1-dependent innate immune genes (*clec-65*, *clec-4*, *irg-1*, *ilys-2*, and *abf-2*) in wild-type and *atfs-1(gk3094)* worms. **(B)** Representative fluorescence images of the *irg-1::GFP* reporter in wild-type and *atfs-1(gk3094)* worms fed OP50 or WK. Scale bars: 100 μm. **(C)** Quantification of *irg-1::GFP* fluorescence intensity shown in panel B (n ≥ 10). **(D)** Representative fluorescence images of the *ilys-2::GFP* reporter in wild-type and *atfs-1(gk3094)* worms fed OP50 or WK. Scale bars: 100 μm. **(E)** Quantification of *ilys-2::GFP* fluorescence intensity shown in panel **(D)** (n ≥ 10). All data are presented as mean ± SEM of three independent experiments performed in triplicate (**P < 0.01, one-way ANOVA).

### *Weissella koreensis* enhances the resistance of mice to PA14 infection and upregulates the transcription of ATF5-associated UPR^mt^ genes in mice

Having demonstrated that *Weissella koreensis* (WK) can enhance host innate immunity through the UPR^mt^ in nematodes, we next sought to determine whether this protective effect could be observed in mammals. C57BL/6J mice were randomly assigned to WK-treated (1.0 × 10^9^ colony-forming units [CFU] per mouse) and saline vehicle control groups and received daily oral gavage for 4 consecutive weeks (**Figure 5A**). After the pretreatment, the mice were challenged with *P. aeruginosa* PA14 (1.0 × 10 (6) CFU per mouse). The survival rate of the WK-treated mice was higher than that of the control group (**Figure 5B**). In parallel, the lung tissues of independent infected groups were collected for histological analysis (hematoxylin and eosin [H&E] staining), CFU enumeration per lung, and qRT-PCR analysis. Consistent with the survival advantage, WK treatment attenuated pulmonary inflammation and reduced lung bacterial burden compared with the controls (**Figures 5C, D**). Meanwhile, in order to probe the underlying molecular mechanism, we measured the mRNA expression levels of *Atf5* and UPR^mt^-related genes (*Hspd1*, *Hspa9*, *Lonp1*, *Yme1l1*) in lung tissues. Compared to the controls, the levels of *Atf5* and UPR^mt^-related genes were upregulated in the WK group (**Figures 5E, F**). Collectively, these results indicate that WK-mediated resistance to pulmonary infection in mice is accompanied by the transcriptional upregulation of the *Atf5*-associated UPR^mt^ program.

**Figure 5:**
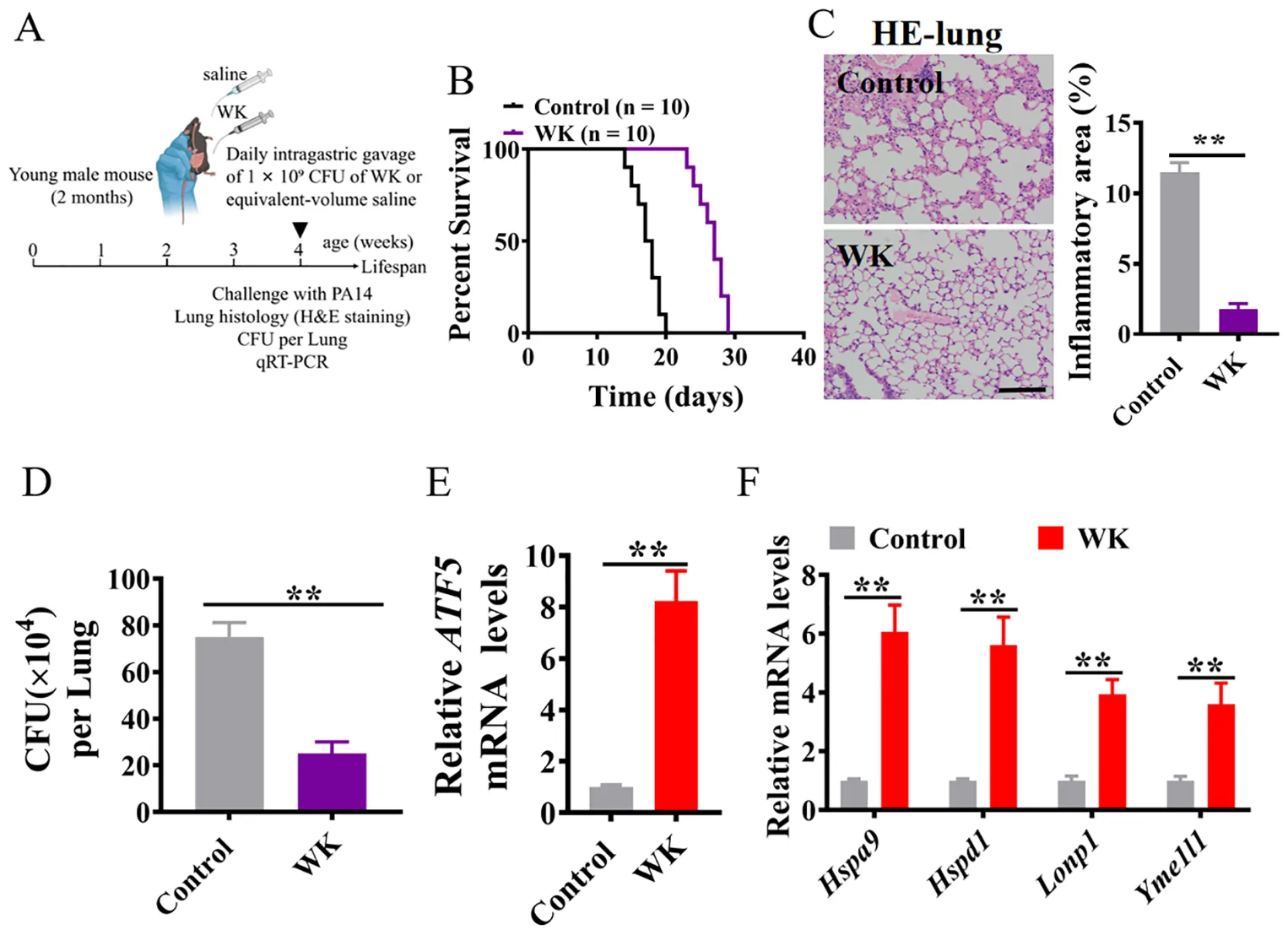
*Weissella koreensis* enhances the resistance of mice to PA14 infection and upregulates the transcription of ATF5-associated UPR^mt^ genes in mice. **(A)** Experimental design schematic in young male C57BL/6J mice pretreated with daily oral gavage of WK (1.0 × 10^9^ CFU per mouse) or saline vehicle for 4 consecutive weeks before intranasal PA14 challenge (1.0 × 10^6^ CFU per mouse). **(B)** Kaplan-Meier survival curves of mice in the saline vehicle and WK groups following PA14 infection (*P < 0.05;log-rank test). **(C)** Representative H&E-stained lung sections and the corresponding quantification of the inflammatory area. Scale bars: 100 μm. (**P < 0.01, unpaired t-test). **(D)** Pulmonary bacterial burden at 48 h after infection, expressed as CFU per lung. (**P < 0.01, unpaired t-test). **(E)** Relative *Atf5* mRNA expression in murine lung tissues (**P < 0.01, unpaired t-test). **(F)** W Relative mRNA expression of canonical murine UPR^mt^-related genes (*Hspd1*, *Hspa9*, *Lonp1*, *Yme1l1*) in lung tissues. Data are presented as mean ± SEM of three independent experiments, each performed in triplicate (**P < 0.01, unpaired *t*-test).

## Discussion

In recent years, the application of probiotics in modulating host immunity and extending health span has garnered extensive attention. The *Weissella* genus has been reported to exhibit diverse health-promoting effects, including anti-tumor effects (17), anti-obesity properties (18, 19, 32), lifespan-extending capabilities (20) and immune-regulating effects (21, 33). For example, *Weissella cibaria* and *Lactobacillus reuteri* attenuated fat accumulation and fatty liver in obese mice (32). *Weissella cibaria* has also been reported to enhance natural killer cell activity in nondiabetic subjects (33). *Weissella koreensis* (WK), a species within the genus *Weissella*, has primarily been investigated for its fermentation characteristics (23). However, accumulating evidence suggests that WK may also exert broader health-promoting effects. For example, WK has been reported to extend the basal lifespan of *C. elegans (*20). Moreover, it also plays a vital role in combating obesity. *W. koreensis* OK1–6 prevents adipogenesis and obesity by suppressing key lipogenic genes (C/EBPα, SREBP1, FAS) (19). Kimchi fermented with *W. koreensis* OK1–6 ameliorates obesity in high-fat diet-fed mice (18). However, the specific role of WK in host immune regulation and the underlying molecular mechanism remains largely undefined, requiring further validation. Here, we identify *Weissella koreensis* as a novel functional probiotic capable of enhancing host innate immunity against bacterial infection. We demonstrate that WK protects *C. elegans* against the pathogen PA14 by significantly reducing the intestinal bacterial burden. Mechanistically, we find WK enhances host antibacterial immunity through an ATFS-1-dependent UPR^mt^ response in *C. elegans* and is associated with transcriptional upregulation of the homologous ATF5-associated UPR^mt^ program in mice (**Figure 6**). Collectively, our findings establish that WK confers protection against bacterial pathogens by upregulating mitochondrial protective genes and innate immune genes (such as those encoding antimicrobial peptides), thereby preserving mitochondrial homeostasis and augmenting host-directed innate defenses.

**Figure 6:**
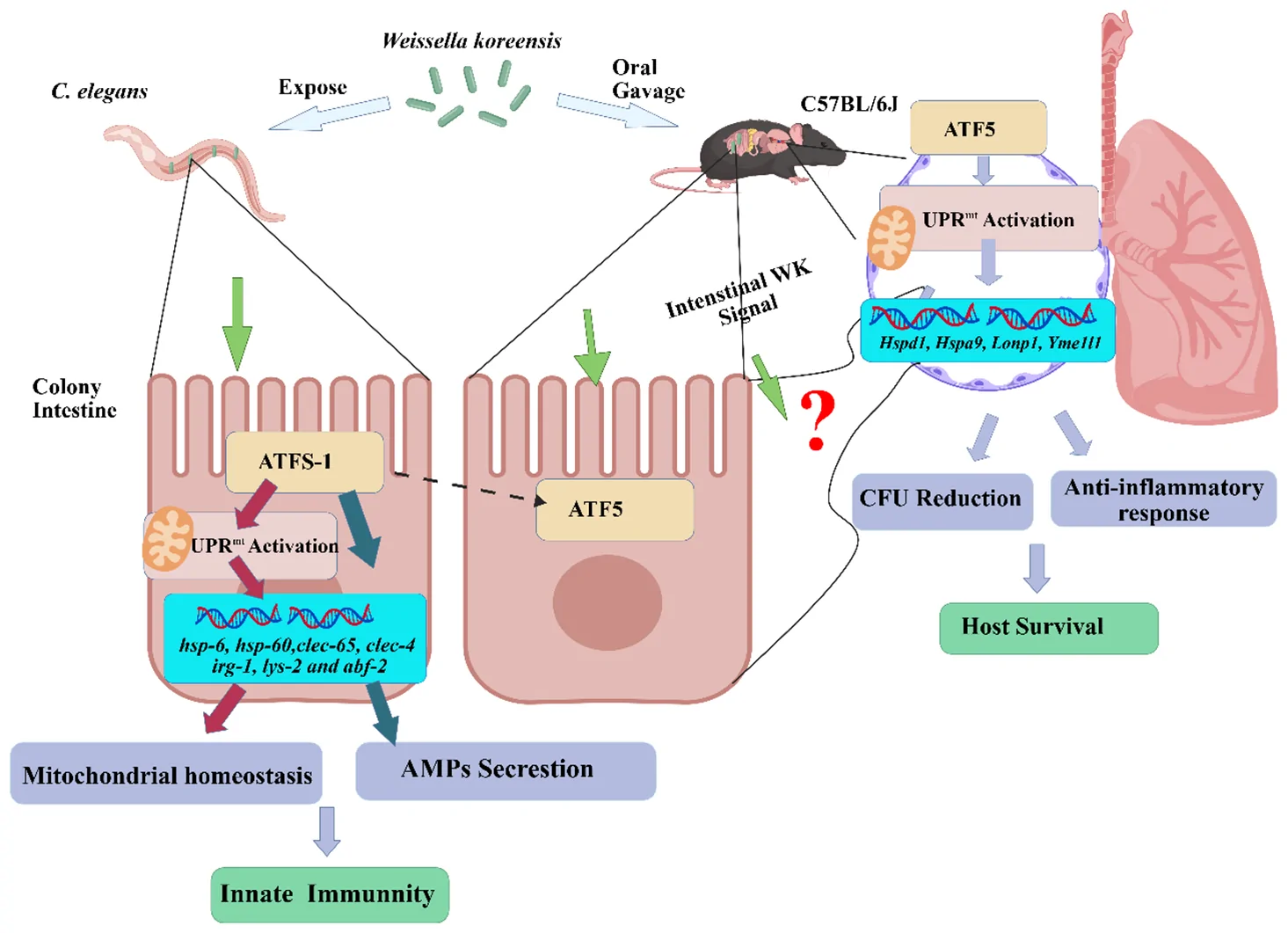
Proposed model for *Weissella koreensis*-mediated host defense in *C. elegans* and mice. Created with BioGDP.com (34).

To dissect the molecular mechanism by which WK enhances immunity, we conducted genetic screening and found that its immune-enhancing eflect is dependent on the transcription factor ATFS-1, but not on other canonical immune-related pathways, such as the DAF-2/DAF-16 axis (5), p38 MAPK/PMK-1 pathway (29), ERK MAPK/MPK-1 (8), UPR^ER^ XBP-1 (10). This finding is consistent with the established role of UPR^mt^ in coordinating mitochondrial protein homeostasis and innate immune defense during pathogen infection (12). In *C. elegans*, mitochondrial stress promotes ATFS-1-dependent transcription of mitochondrial chaperones and proteases to restore protein homeostasis, while also inducing antimicrobial and other innate immune effector genes. WK specifically increased the mRNA levels of *atfs-1* and activated the UPR^mt^ reporter *hsp-60::GFP*. WK also enhanced the expression of ATFS-1 associated immune eflector genes, including *clec-65*, *clec-4*, *irg-1*, *ilys-2*, and *abf-2*, together with mitochondrial protective genes (*hsp-6 and hsp-60*). Notably, these transcriptional responses were markedly reduced or abolished in *atfs-1* mutants, further supporting the central role of ATFS-1 in WK-induced host defense. Collectively, these results indicate that WK coordinates immune defense and mitochondrial homeostasis through ATFS-1-mediated UPR^mt^ activation, thereby strengthening host resistance to bacterial infection.

The mechanism underlying this immune-enhancing effect appears to involve coordinated regulation of mitochondrial homeostasis and antimicrobial defenses. Probiotics can produce bioactive metabolites, including exopolysaccharides (EPS) and organic acids, which possess strong antioxidant and immunomodulatory properties (35, 36). The concurrent observation of reduced ROS levels, elevated ATP production, and sustained UPR^mt^ activation in our study initially appears paradoxical, as UPR^mt^ is classically triggered by mitochondrial stress characterized by high ROS and bioenergetic deficits. One possible explanation is that WK induces a mild mitochondrial stress response that activates ATFS-1-dependent UPR^mt^ signaling, followed by an adaptive increase in mitochondrial chaperones and antioxidant capacity. The subsequent marked upregulation of mitochondrial chaperones and endogenous antioxidant defenses optimizes respiratory chain efficiency. Consequently, the suppressed ROS accumulation and elevated ATP production we observed represent the adaptive steady state resulting from the resolved UPR^mt^ response, rather than the initial triggering event. Another possibility is that sustained exposure to WK-derived metabolites may represent one possible class of microbial signals, allowing WK to bolster host defense without causing pathological mitochondrial dysfunction. Importantly, we did not directly determine whether bacterial surface components, secreted products, or microbial metabolites mediate this response. Identifying the WK-derived factors that initiate ATFS-1 activation will require bacterial fractionation, metabolomic profiling, and functional validation of candidate components. Our findings also distinguish the observed protection from a purely direct antibacterial effect. Although *Weissella* species have been reported to antagonize pathogens through bacteriocins or hydrogen peroxide secretion (37), our data indicates that WK enhances host defense through a host-directed mechanism. In particular, disruption of ATFS-1 abolished the protective effect of WK in *C. elegans*, supporting a requirement for this host transcription factor in WK-mediated antibacterial protection. Thus, while a direct antimicrobial contribution of WK cannot be excluded, the current findings support a central role for host ATFS-1-dependent UPR^mt^ signaling in the protective phenotype observed in *C. elegans*.

This protective phenotype was observed in both *C. elegans* and mice, although the underlying mammalian mechanism remains to be established. In mice, oral administration of WK increased *Atf5* mRNA expression and the expression of associated mitochondrial protective genes and reduced pulmonary bacterial burden and inflammation following PA14 infection. These findings raise the possibility that signals generated following intestinal WK exposure may influence distal pulmonary immunity through the gut-lung axis. Pulmonary inflammation was further quantified by measuring the inflammatory area in H&E-stained lung sections, providing quantitative support for the histological findings. However, because we did not directly trace the intestinal signal or establish a causal link between WK exposure and pulmonary ATF5 activation, this gut-lung connection remains a working model rather than a demonstrated mechanism.

Several questions remain unresolved. The specific WK-derived factor(s) responsible for engaging the host mitochondrial stress response have yet to be identified. Although bacterial surface components, secreted products, and microbial metabolites are plausible candidates, the present study does not distinguish among these possibilities. Future studies combining bacterial fractionation, metabolomic profiling, and functional testing of candidate factors will be important for identifying the microbial signal that links intestinal WK exposure to ATFS-1 activation. In addition, our loss-of-function experiments show that ATFS-1 is required for WK-mediated protection in *C. elegans*, but whether increased ATFS-1 activity is sufficient to reproduce this phenotype remains unclear. This question could be addressed using *atfs-1* gain-of-function or overexpression models. Similarly, although the murine findings support a conserved protective phenotype, protein-level validation and mammalian loss-of-function studies are needed to determine whether the ATF5-associated UPR^mt^ pathway is mechanistically required for WK-mediated protection in mice. Although pulmonary inflammation was quantitatively assessed histologically, we did not profile specific inflammatory genes or cytokines. The murine experiments included a WK-treated group and a saline vehicle control group, with both groups subjected to PA14 challenge, allowing us to directly assess the effect of WK pretreatment on host resistance to infection. However, the absence of an uninfected control group limited our ability to determine whether WK treatment altered basal pulmonary inflammatory status or baseline expression of *Atf5* and UPR^mt^-related genes before PA14 challenge. Consequently, it remains unclear whether the observed *Atf5* upregulation and UPR^mt^-related transcriptional changes reflect a direct effect of WK treatment or a secondary response to PA14 infection. Future studies incorporating uninfected controls, pre-infection baseline measurements, comprehensive inflammatory profiling, and mechanistic interrogation of the gut-lung axis will help distinguish the effects of WK treatment from those associated with PA14 infection.

Collectively, our present study identifies *Weissella koreensis* as a functional probiotic that enhances host antibacterial defense through an ATFS-1-dependent mitochondrial stress-response pathway in *C. elegan*s, with a potentially related transcriptional response observed in mice. These findings broaden our understanding of the immunomodulatory functions of WK and extend its potential application beyond its traditional role in food fermentation toward probiotic-based immune intervention. Future investigations should employ metabolomics to isolate and identify the specific WK-derived effector molecules responsible for UPR^mt^ activation.

## Materials and methods

### Worm strains and bacterial culture

Worms were maintained and propagated on nematode growth medium (NGM) plates coated with *E. coli* OP50 or *Weissella koreensis* under standard conditions (2, 38). The bacterial cells were harvested by centrifugation, resuspended, and standardized to an optical density at 600 nm (OD_600_) of 0.9 for subsequent experiments. The following worm strains were obtained from the Caenorhabditis Genetics Center (CGC at the University of Minnesota, USA) and maintained according to standard protocols (39): N2 Bristol wild-type, KU25 *pmk-1(km25)*, SD184 *mpk-1(n2521)*, CB1370 *daf-2(e1370)*, SJ17 *xbp-1(zc12)*, VC3201 *atfs-1(gk3094)*, SJ4058 (*hsp-60p::GFP*), OP675 (*atfs-1::GFP*), AU133 (*irg-1::GFP*), OP675 *atfs-1::GFP*, and CB6725 (*ilys-2::GFP*). *Weissella koreensis* was obtained from the Lactic Acid Bacteria and Bifidobacterium Strain Resource Center (LABCC), Inner Mongolia Agricultural University.

### Generation of genetic crosses

Standard genetic crosses were performed to introduce GFP reporter transgenes into the *atfs-1(gk3094)* mutant background. Wild-type males were generated by heat shock at 30 °C for 4 h and mated with homozygous hermaphrodites carrying the *atfs-1(gk3094)* deletion allele. Heterozygous male progeny from the first cross were subsequently mated with hermaphrodites carrying the integrated transcriptional reporters *hsp-60*::GFP, *irg-1*::GFP, or *ilys-2*::GFP. GFP-positive cross-progeny were isolated individually and allowed to self-fertilize. In the F2 generation, GFP-expressing worms were again singled onto individual NGM plates. Homozygosity for the *atfs-1(gk3094)* allele was confirmed in the F3 generation by single-worm PCR using allele-specific primers spanning the deletion region, and stable transmission of the reporter transgene was confirmed by fluorescence microscopy of the progeny. The resulting homozygous double-mutant/reporter lines, *atfs-1(gk3094)*; *hsp-60*::GFP, *atfs-1(gk3094)*; *irg-1*::GFP, and *atfs-1(gk3094); ilys-2*::GFP, were established and backcrossed before subsequent phenotypic and fluorescence imaging analyses.

### Construction of GFP-labeled *Weissella koreensis*

GFP-labeled WK was generated by introducing a commercially obtained GFP-expressing plasmid (pBBR1MCS5-Tac-EGFP) into WK through bacterial transformation. GFP expression was confirmed by fluorescence microscopy. The resulting GFP-labeled WK was used for intestinal colonization analysis in *C. elegans*.

### Infection assay

*E. coli* OP50, *S. enterica* SL1344, *E. faecalis* ATCC 29212, and *P. aeruginosa* PA14 were grown overnight in LB broth at 37 °C with shaking with 180 rpm. *S. aureus* NCTC8325 was grown overnight in tryptic soy broth (TSB, BD, Sparks, MD) under identical conditions. All infection assays were performed on NGM agar plates. Notably, after the PA14 bacterial suspension had dried on the plates, the seeded agar was incubated at 37 °C for 16–18 h to induce virulence factor production, followed by a 3-hour attenuation at 25 °C prior to use. Synchronized populations of worms were cultivated on *E. coli* OP50 or *Weissella koreensis* at 20 °C until the young adult stage. Subsequently, approximately 40–60 worms were transferred to individual NGM agar plates seeded with *S. enterica* SL1344, *S. aureus* NCTC8325, or *P. aeruginosa* PA14, and incubated at 25 °C. The number of living worms was recorded at 12-hour intervals. Individuals that failed to respond to gentle mechanical stimulation were scored as dead (31, 40–42). Each assay was performed using three replicate plates, and all experiments were repeated in three independent biological trials.

### Quantification of intestinal bacterial burden

Synchronized populations of worms were cultivated on *E. coli* OP50 or WK at 20 °C. Upon reaching the young adult stage, they were shifted to NGM plates precoated with *P. aeruginosa* PA14 and transferred at 25 °C for 48 h (43, 44). To remove any surface-bound PA14, the nematodes were thoroughly washed three times with sterile M9 buffer, with each washing step lasting approximately 1 minute to remove surface-bound bacteria before homogenization. Lysis was performed by homogenizing ten worms in 50 μL of PBS containing 0.1% Triton. Finally, the homogenates were subjected to 10-fold serial dilutions in sterile water, spotted onto ampicillin-supplemented LB agar (100 μg/mL), and incubated for 48 hours at 37 °C to determine PA14 colony-forming units (CFUs). Each assay was performed using three replicate plates, and all experiments were repeated in three independent biological trials.

### Fluorescence microscopy analysis

*C. elegans* strains carrying *irg-1::GFP*, *ilys-2::GFP*, *atfs-1::GFP* and *hsp-60::GFP* were synchronized. Eggs were plated onto NGM plates, and once the worms reached the young adult stage on *E. coli* OP50- or WK-seeded NGM plates, they were transferred to fresh NGM plates for a 48-hour incubation. Fluorescent images were obtained using a Zeiss Axioskop 2 Plus fluorescence microscope (Carl Zeiss, Jena, Germany), and relative fluorescence intensity was quantified using ImageJ software (NIH) (45). Each condition included three plates (approximately 35–40 worms per plate) across three independent trials.

### Measurements of ROS

ROS levels were measured using the probe 2,7-dichlorodihydrofluorescein diacetate (DCF-DA), following the protocol as previously described (46–48). Synchronized L1 larvae were placed onto NGM plates seeded with either *E. coli* OP50 or WK, and cultured at 20 °C. Upon reaching the young adult stage, about 5000 worms from both the OP50 and WK groups were collected and washed with M9 buffer to eliminate residual bacteria. Fluorescence intensity was quantified, and total protein concentrations were determined using a bicinchoninic acid (BCA)Protein Assay Kit (Beyotime Biotechnology, Shanghai, China; Cat. No. P0012S)) according to the manufacturer’s instructions. ROS fluorescence intensity was normalized to the corresponding total protein levels. All experiments were performed in three independent biological replicates.

### ATP determination

To evaluate intracellular ATP levels, synchronized *C. elegans* L1 larvae were cultured at 20 °C on NGM plates seeded with either *E. coli* OP50 or WK. Upon reaching the young adult stage, about 5000 worms were collected and washed with M9 buffer. ATP quantification was subsequently performed using a commercially available assay kit (CellTiter-Glo; Promega, Fitchburg, WI, USA) following the manufacturer’s protocol (49). The experiment was independently repeated three times.

### Quantitative real-time PCR

Synchronized *C. elegans* were maintained on *E. coli* OP50 or WK lawns until reaching the young adult stage. The nematodes were exposed to *P. aeruginosa* PA14 for 24 h. After the incubation period, the nematodes were flash-frozen in liquid nitrogen and total RNA was extracted using TRIzol reagent as per the manufacturer’s instructions. The extracted RNA was then reverse-transcribed into cDNA using random primers and SuperScript II (Invitrogen). Subsequently, quantitative real-time PCR was performed on an Applied Biosystems Prism 7000 Sequence Detection System (Applied Biosystems, Foster City, CA, USA) utilizing SYBR Premix Ex Taq™ (Takara, Dalian, China). The *pmp-3* gene was employed as the internal control for normalization, as previously described (50, 51). Each experiment was conducted in three independent biological replicates, and the specific primer sequences utilized are detailed in **Supplementary Table S1**.

### Animal experiments

Male C57BL/6J mice (2 months of age) were obtained from Zunyi Medical University. Male mice were exclusively used in this initial proof-of-concept study to minimize potential biological variability associated with hormonal fluctuations during the estrous cycle, which may influence systemic immune responses and gut microbiota composition. Mice were randomly allocated to a vehicle control group or a WK treatment group. The WK-treated group received a daily oral gavage of WK (1.0 × 10^9^ CFU per mouse), whereas the control group received an equivalent volume of sterile saline for 4 consecutive weeks. Following the 4-week pretreatment period, mice were challenged with PA14 by intranasal administration. Each mouse received 10 μL of a PA14 (1.0 × 10^6^ CFU) via nasal drops (10). Endpoint lung sampling, survival monitoring, and bacterial burden determination were conducted using two independent cohorts of mice, following previously described murine infection and physiological assessment protocols. In the survival cohort, mice (n = 10 per group) were monitored after PA14 challenge, and survival was recorded at designated time intervals. In the molecular and histological analysis cohort, biologically independent mice (n = 8 per group) were euthanized by cervical dislocation at 48 h after PA14 challenge for lung tissue collection. For pulmonary bacterial burden determination, one lung from each mouse was aseptically collected, placed in 1 mL of sterile PBS, and homogenized. The homogenates were serially diluted 10-fold and plated onto LB agar plates, followed by incubation at 37 °C for 18–24 h. Viable *P. aeruginosa* PA14 colonies were enumerated, and the bacterial burden was expressed as CFU per lung. The remaining lung tissues from the same animals were either fixed in 10% neutral buffered formalin for H&E staining to assess pulmonary inflammation or processed for RNA extraction and subsequent quantitative real-time PCR (qRT-PCR) analysis. The procedure was performed by highly trained personnel in strict accordance with the American Veterinary Medical Association (AVMA) Guidelines for the Euthanasia of Animals to minimize animal suffering. All animal experimental procedures and welfare considerations were reviewed and approved by the Animal Research Ethics Committee of Zunyi Medical University (Approval No. ZMU21-2405-006). The present animal study was performed following the recommendations outlined in the ARRIVE guidelines (52).

### H&E staining

Left lung tissues collected from the vehicle and WK treatment groups were subjected to H&E staining to determine the extent of inflammatory infiltration. The excised lungs were fixed in 10% neutral formalin, dehydrated through a graded ethanol series, and embedded in paraffin. Sliced 5μm tissue sections were baked for 1 h at 60 °C, cleared with xylene, and rehydrated through an ethanol gradient. Tissue architecture and inflammatory responses were evaluated microscopically and quantitatively analyzed according to established histological assessment protocols (53).

### Statistics

Data were presented as mean ± standard error of the mean (SEM). Graphs were generated with GraphPad Prism 9.0 software (GraphPad, San Diego, CA, USA). Statistical analyses for all data except for survival assays were carried out using Student’s *t*-test (unpaired, two-tailed) or analysis of variance (ANOVA) after testing for normal distribution and homogeneity of variances. Survival data were analyzed using the log-rank (Mantel-Cox) test (54). P value < 0.05 was considered statistically significant.

## Data Availability

The original contributions presented in the study are included in the article/**Supplementary Material**. Further inquiries can be directed to the corresponding authors.
