# Supplementary material for: Weissella koreensis regulates mitochondrial protective genes and innate immune genes to enhance anti-bacterial immunity
Source: Front Immunol. 2026 Oct 21;17:1998836. doi: 10.3389/fimmu.2026.1998836 (PMC13730957; doi:10.3389/fimmu.2026.1998836)
Supplement: Supplementary file 1 [file SupplementaryFile1.docx]

**Figure S1** WK did not influence the pumping rate in WT and *atfs-1(gk3094)* mutant worms. NS (no significance).

**Figure S2** Heat-killed WK did not increase the expression level of *hsp-60::GFP*. NS (no significance). Scale bar: 100 μm.
